# Myositis autoantibodies in Korean patients with inflammatory myositis: Anti-140-kDa polypeptide antibody is primarily associated with rapidly progressive interstitial lung disease independent of clinically amyopathic dermatomyositis

**DOI:** 10.1186/1471-2474-11-223

**Published:** 2010-09-28

**Authors:** Eun Ha Kang, Ran Nakashima, Tsuneyo Mimori, Jinhyun Kim, Yun Jong Lee, Eun Bong Lee, Yeong Wook Song

**Affiliations:** 1 Division of Rheumatology Department of Internal Medicine, Seoul National University Bundang Hospital, Sungnam, Korea; 2 Department of Rheumatology and Clinical Immunology, Kyoto University Graduate School of Medicine, Kyoto, Japan; 3 Division of Rheumatology Department of Internal Medicine, Seoul National University College of Medicine, Seoul, Korea

## Abstract

**Background:**

To investigate the association between myositis autoantibodies and clinical subsets of inflammatory myositis in Korean patients.

**Methods:**

Immunoprecipitation was performed using the sera of classic polymyositis (PM) (n = 11) and dermatomyositis (DM) (n = 38) patients who met the Bohan and Peter criteria for definite inflammatory myositis. A panel of defined myositis autoantibodies was surveyed to investigate the association between each autoantibody and clinical subsets of inflammatory myositis.

**Results:**

Either MSAs, anti-p140, or anti-p155/140 antibodies were found in 63.3% (31/49) of the study subjects. Anti-140-kDa-polypeptide (anti-p140) (18.4%, 9/49) and anti-155/140-kDa polypeptide (anti-p155/140) (16.3%, 8/49) antibodies were the most common, followed by anti-Mi2 (14.3%, 7/49), anti-ARS (12.2%, 6/49) and anti-SRP (2.0%, 1/49) antibodies. All MSAs and anti-p140 and anti-p155/140 antibodies were mutually exclusive. Anti-p140 (23.7%, 9/38), anti-p155/140 (21.1%, 8/38), and anti-Mi2 (18.4%, 3/38) antibodies were found exclusively in DM patients. Anti-p140 antibody was associated with rapidly progressive interstitial lung disease (ILD) (p = 0.001), with a sensitivity of 100.0% (4/4) and a specificity of 85.3% (29/34) in DM patients. Anti-p155/140 antibody was associated with cancer-associated DM (p = 0.009), with a sensitivity of 55.6% (5/9) and a specificity of 89.7% (26/29). Cancer-associated survival was significantly worse when anti-p155/140 antibody was present (19.2 ± 7.6 vs. 65.0 ± 3.5 months, p = 0.032). Finally, anti-ARS antibodies were associated with stable or slowly progressive ILD in PM and DM patients (p = 0.005).

**Conclusions:**

Anti-p140 and anti-p155/140 antibodies were commonly found autoantibodies in Korean patients with inflammatory myositis. Despite the lack of clinically amyopathic DM patients in the study subjects, a strong association was observed between anti-p140 antibody and rapidly progressive ILD. Anti-p155/140 antibody was associated with cancer-associated myositis and poor survival.

## Background

Polymyositis (PM) and dermatomyositis (DM) are systemic autoimmune diseases in which muscles are the primary target of immune-mediated inflammation. In addition to muscular inflammation and dysfunction, the systemic complications of PM and DM involve vessels, joints, the gastrointestinal tract, cardiac tissues, and lungs [1]. In particular, damage to lung parenchyma, which manifests as interstitial lung disease (ILD), and accompanying malignancies are the major prognostic factors that contribute to mortality in PM and DM patients [2,3]. On the other hand, amyopathic dermatomyositis (ADM) is a condition in which the typical skin manifestations of DM develop without muscle involvement, and it constitutes the clinical spectrum of inflammatory myositis together with PM and DM [4]. Clinically amyopathic dermatomyositis (CADM) is an extended concept of ADM in which no muscle weakness is observed with or without subclinical evidence of muscle inflammation on laboratory, electrophysiological, and/or radiographic evaluations [5]. Treatment-resistant rapidly progressive interstitial lung disease (ILD) has been reported to cluster in ADM/CADM patients [5-7], and appreciable clinical significance has been conferred upon ADM and/or CADM (ADM/CADM).

As in other connective tissue diseases, PM and DM are characterized by autoantibodies to various cellular components. Some of these autoantibodies are found specifically in PM and DM patients (referred to as myositis-specific autoantibodies, MSAs) or in myositis overlap syndrome patients (myositis-associated autoantibodies, MAAs). The MSAs tend to be mutually exclusive and are associated with certain clinical subsets [8], which renders MSAs as useful tools to classify clinical subgroups. The most striking association found to date concerns the association between anti-aminoacyl-tRNA synthetase (anti-ARS) antibodies and the presence of ILD [2]. In recent years, novel autoantibodies have been identified in inflammatory myositis, such as, anti-140-kDa polypeptide (anti-p140) [9] and anti-155/140-kDa polypeptide (anti-p155/140) antibodies [10,11]. Because these antibodies have yet to be extensively studied in non-myositis populations to assure their specificity for myositis and because the presence of anti-p140 antibodies has been largely limited to CADM patients who do not have clinical muscle symptoms [9,12,13], it may be currently inappropriate to classify anti-p140 and anti-p155/140 antibodies as MSAs. However, associations between these novel antibodies and distinctive clinical subsets have been found in adult inflammatory myositis patients; associations between anti-p140 antibody and CADM-associated ILD [9,12,13] and between anti-p155/140 antibody and cancer-associated myositis are such examples [10-12,14-16]. The clinical usefulness of these autoantibodies has well been recognized as diagnostic markers that could potentially alter disease outcomes by facilitating early diagnosis and treatment. However, clinical implications regarding these novel antibodies in adult PM and DM patients have been limited to a few ethnic cohorts [9-16]. Given that the phenotypes and severities of connective tissue diseases are often influenced by genetic background [17,18], extended observations of other racial groups are mandatory. In the present study, we investigated the panel of defined autoantibodies including MSAs, MAAs, anti-p140, and anti-p155/140 antibodies in the sera of Korean inflammatory myositis patients with the intention to classify clinical subsets of these patients based on the presence of myositis autoantibodies and to refine the relationships between these antibodies and disease manifestations.

## Methods

### Patients and sera

Forty nine serum samples (n = 11 for PM, n = 38 for DM) were available from seventy-five patients (n = 20 for PM, n = 55 for DM) consecutively diagnosed as having definite inflammatory myositis according to the Bohan and Peter criteria [19] from March 1993 to November 2007 at the Rheumatology Clinic of Seoul National University Hospital. The remaining twenty-six sera had been examined for the presence of certain MSAs in our previous study [20], but were not available for the current study. DM was classified when heliotrope rash, Gottron's sign, and/or Gottron's papule were present. Patients with myositis overlap syndrome met both the above criteria and the criteria for another defined connective tissue disease. Patients with juvenile DM (age ≤ 18), ADM/CADM (classified according to the criteria by Sontheimer [5]), or inclusion body myositis (diagnosed by the presence of typical inclusions on a stained muscle biopsy under the light microscopy) were not included.

Clinical information regarding disease manifestations, laboratory data, radiographic data, and the presence of internal malignancies was obtained by medical chart review. Chest radiography (CXR) and/or high resolution computed tomography (HRCT) were performed at baseline and repeated every 12 months or when they had new onset respiratory symptoms. Patients were diagnosed as having ILD based on the radiographic evidence in CXR and/or HRCT findings. Rapidly progressive ILD was defined to be present when ILD showed radiographic deterioration causing hypoxia within one month from respiratory symptom onset. All patients underwent cancer screening, including chest radiography, computed tomography for abdomen and pelvis, and endoscopy for stomach and colon. Breast and gynecologic examinations were done for female patients. Patients negative at initial cancer screening were re-examined whenever suspected for malignancy during follow-up. Cancer-associated myositis was identified when cancer was detected within 3 years of myositis diagnosis [14]. This study was approved by the Institutional Review Board of Seoul National University Hospital (#1002-003-308) and informed consent was obtained from study participants.

### Immunoprecipitation assay

Immunoprecipitation was performed using extracts of HeLa cells, as previously described [13,21]. For RNA analysis, 2 mg of protein A-Sepharose CL (GE Healthcare, Sweden) incubated with 10 μl of sera in 500 μl immunoprecipitation buffer (IPP; 10 mM Tris-Cl, 500 mM NaCl, 0.1% Nonidet P-40, pH 8.0) was washed 4 times with 500 μl IPP, resuspended in 400 μl NET-2 buffer (50 mM Tris-HCl at pH 7.5, 150 mM NaCl, 0.05% NP-40) and mixed with 100 μl of HeLa cell extracts (6 × 10^6 ^cell equivalent per sample) for 2 hrs at 4°C. The Sepharose was then collected, washed 4 times with NET-2 buffer, and then resuspended in 300 μl of NET-2 buffer. To extract bound RNA, 30 μl of 3.0 M sodium acetate, 30 μl of 10% sodium dodecyl sulfate (SDS), 2 μl of carrier yeast tRNA (10 mg/mL; Sigma, St. Louis, USA) and 300 μl of phenol:chloroform:isoamyl alcohol (50:50:1, containing 0.1% 8-hydroxyquinolone) were added. After ethanol precipitation, RNAs were resolved in 7 M urea-10% polyacrylamide gel, which was subsequently silver-stained (Bio-Rad, Hercules, USA). MSA and MAA reactivities was assessed using reference sera containing autoantibodies to histidyl tRNA synthetase (Jo-1), glycyl tRNA synthetase (EJ), asparaginyl tRNA synthetase (KS), isoleucyl tRNA synthetase (OJ), threonyl tRNA synthetase (PL-7), alanyl tRNA synthetase (PL-12), signal recognition particle (SRP), U_1 _RNP, Ro, La, Ku, Sm, or Th/To.

For polypeptide analysis, 2 mg of IgG-coated protein A-Sepharose CL in 400 μl IPP was mixed with 100 μl of radiolabeled HeLa cell extract for 2 hrs at 4°C. After 4 washes with IPP, the beads were spun down, boiled for 5 min in 30 μl of sample buffer, and then fractionated by 10% SDS-polyacrylamide gel electrophoresis. After autoradiography, autoantibody reactivity was assessed versus three reference sera, containing anti-p140, anti-p155/140, or anti-Mi2 antibodies. The presence of anti-p140 or anti-p155/140 antibodies was defined when apparent protein precipitates were found either at 140-kDa (anti-p140) or at 155/140-kDa (anti-p155/140) which match the reference sera. Studies to further confirm the specificities of these antibodies were not performed.

### Statistical analysis

Continuous values are represented as means ± standard deviation (SD). Statistical analysis was performed using the Mann Whitney U test and the chi-square test (or Fisher's exact test if appropriate) to compare continuous and categorical variables, respectively. Cumulative survival rates were determined using the Kaplan-Meier method and the log-rank test; survival times were presented as means ± standard error (SE). SPSS (SPSS Inc., Chicago, IL) was used throughout, and two-sided p-values of < 0.05 were considered statistically significant.

## Results

### Clinical characteristics of the study subjects

The study subjects showed a slight female predominance (61.2% among all study subjects) with a mean age of 45.4 ± 14.6 (mean ± SD) years (table 1). Patients were followed for 59.1 ± 51.9 months after the diagnosis of inflammatory myositis. ILD rates were 18.2% (2/11) in the PM group and 31.6% (12/38) in the DM group. All rapidly progressive ILD developed in DM patients (n = 4). Cancer was present in 12 patients, 11 of whom were classified as cancer-associated myositis (table 2). There were 3 myositis overlap cases (n = 1 for DM/systemic sclerosis, n = 1 for DM/systemic lupus erythematosus (SLE), n = 1 for PM/SLE). Ten patients died during follow-up of 14.6 ± 19.5 months; 6 died of cancer, 3 of ILD, 1 of bacterial pneumonia, which suggests that cancer and ILD are the two most important prognostic factors of myositis. Overall, no significant clinical difference was observed between the PM and DM groups.

**Table 1:** Demographic and clinical data of PM and DM patients

	PM (n = 11)	DM (n = 38)	Total (n = 49)
Age at diagnosis (years)†	51.1 ± 15.8	43.7 ± 13.9	45.4 ± 14.6
Gender (F:M)	7:4	23:15	30:19
Disease duration (months)†	44.2 ± 46.9	62.9 ± 53.0	59.1 ± 51.9
Clinical manifestation, n (%)			
Fever	3 (27.3)	11 (28.9)	14 (28.6)
Raynaud's phenomenon	2 (18.2)	6 (15.8)	8 (16.3)
Arthralgia	2 (18.2)	14 (36.8)	16 (32.7)
Dysphagia	0 (0)	9 (23.7)	9 (18.4)
Cardiac involvement	2 (18.2)	0 (0)	2 (4.1)
ILD	2 (18.2)	12 (31.6)	14 (28.6)
Malignancy	3 (27.3)	9 (23.7)	12 (24.5)
Myositis overlap	1 (9.1)	2 (5.3)	3 (6.1)

† mean ± SD

DM = dermatomyositis, F = female, ILD = interstitial lung disease, M = male, PM = polymyositis

**Table 2:** Clinical data of 12 patients who had malignancy

	Age (years)	Sex	Diagnosis	ILD	Malignancy (n = 12)			Detected autoantibodies
						
					Time of detection† (months)	Primary site	Cancer-associated myositis	
1	56	F	DM	-	-11	Esophagus	Yes	Anti-p155/140
2	46	F	DM	-	+18	Breast	Yes	Anti-Mi2
3	59	M	DM	-	-1	Stomach	Yes	Anti-p155/140
4	53	F	DM	-	0	Lung	Yes	Anti-p155/140
5	74	F	PM/SLE	+	- 48	Stomach	No	Anti-PL-7
6	57	M	PM	-	0	Liver	Yes	none
7	36	F	DM/SLE	+	-1	Thyroid	Yes	Anti-PL-12
8	36	M	DM	-	0	Lymphoma	Yes	Anti-p155/140
9	50	F	DM	-	-2	Breast	Yes	Anti-p155/140
10	61	M	DM	+	+22	Lymphoma	Yes	Anti-Jo-1
11	68	M	DM	-	+2	Lung	Yes	none
12	65	M	PM	+	0	Stomach	Yes	Anti-Jo-1

† Relative to time of myositis diagnosis.

DM = dermatomyositis, F = female, ILD = interstitial lung disease, M = male, PM = polymyositis, SLE = systemic lupus erythematosus

### Autoantibody frequencies

**Figure 1:**
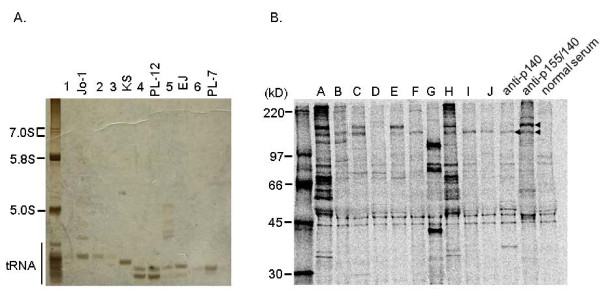
**Representative immunoprecipitation results**. A. tRNA precipitated by patient and prototype sera. Patients 1 - 3 were positive for anti-Jo-1 antibody, patient 4 for anti-PL-12 antibody, patient 5 for anti-EJ antibody, and patient 6 for anti-PL-7 antibody. B. Polypeptides precipitated by patient and prototype sera. Patients B, F, I, and J were positive for anti-p140 antibody, patients C and E for anti-p155/140 antibody, patients A and H for anti-Mi2 antibody, and patient G (= patient 4) for anti-PL-12 antibody.

Immunoprecipitation assays showed that 9 patients had anti-p140 (18.4%), 8 anti-p155/140 (16.3%), 7 anti-Mi2 (14.3%), 6 anti-ARS (12.2%), and 1 anti-SRP (2.0%) antibodies, and that 10 patients had MAAs (figure 1 and table 3). The MSAs, anti-p140, and anti-p155/140 antibodies were found to be mutually exclusive. Anti-p140, anti-p155/140, and anti-Mi2 were exclusively found in DM patients at the prevalence of 23.7% (9/38), 21.1% (8/38), and 18.4% (7/38), respectively. Anti-ARS antibodies included anti-Jo-1 (n = 3), anti-PL-7 (n = 1), anti-PL-12 (n = 1), and anti-EJ (n = 1) antibodies. Of the MAAs, anti-U_1_RNP antibody was most frequently observed (6/49, 12.2%). Anti-Ku, anti-Sm, and anti-Th/To antibodies were not detected. Three cases had autoantibodies with unknown specificities.

**Table 3:** Antibody profiles determined by immunoprecipitation

	PM n = 11 (%)	DM n = 38 (%)	Total n = 49 (%)
Myositis specific antibodies	4 (36.4)	10 (26.3)	14 (28.6)
Anti-ARS antibodies	3 (27.3)	3 (7.9)	6 (12.2)
Anti-Mi2 antibody	0	7 (18.4)	7 (14.3)
Anti-SRP antibody	1 (9.1)	0	1 (2.0)
			
Anti-p140 antibody	0	9 (23.7)	9 (18.4)
Anti-p155/140 antibody	0	8 (21.1)	8 (16.3)
			
Myositis associated antibodies	1 (9.1)	9 (23.7)	10 (20.4)
Anti-U_1 _RNP	0	6 (15.8)	6 (12.2)
Anti-Ro	1 (9.0)	3 (7.9)	4 (8.2)
Anti-La	0	1 (2.6)	1 (2.0)

ARS = aminoacyl tRNA synthetase, DM = dermatomyositis, PM = polymyositis, SRP = signal recognition particle, RNP = ribonucleoprotein

### Associations between autoantibodies and clinical subsets

**Figure 2:**
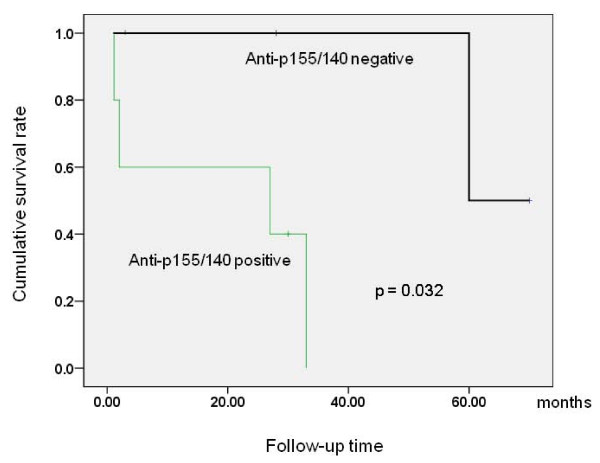
**Cumulative survival rates in cancer-associated dermatomyositis patients according to anti-p155/140 antibody status**.

We then examined whether any associations existed between the myositis autoantibodies and the clinical features of myositis. Table 4 presents the clinical characteristics of 9 patients positive for anti-p140 antibody and table 5 demonstrates distinct clinical subsets associated with anti-ARS, anti-p140, or anti-p155/140 antibodies. Anti-ARS positive patients had a higher frequency of ILD than negative patients (5/6 vs. 9/43, p = 0.005), with a 100% prevalence of ILD in anti-Jo-1 positive patients. One PM patient with anti-PL-7 antibody did not develop ILD but had serositis. Three of 6 anti-ARS positive patients had cancer-associated myositis without statistically significant association (3/6 vs. 8/43, p = 0.117). The clinical phenotype of ILD in the presence of anti-ARS antibody was either slowly progressive or stable. Anti-p140 positive patients also showed a higher frequency of ILD than anti-p140 negative patients (6/9 vs. 8/40, p = 0.011). In particular, rapidly progressive ILD was found to be exclusively associated with the anti-p140 antibody (4/9 vs. 0/40, p = 0.001). The mean survival time (mean ± SE) of patients with rapidly progressive ILD (n = 4) was 10.2 ± 5.8 months, whereas that of those without ILD (n = 45) was 109.6 ± 9.5 months (p = 0.002), which suggest that rapidly progressive ILD is a major prognostic factor. Anti-p155/140 antibody was found to be associated with cancer-associated myositis (5/8 vs. 6/41, p = 0.009). Anti-p155/140 positive cancer patients (n = 5) tended to have poor outcome, showing a shorter survival time than anti-p155/140 negative cancer patients (n = 6) (19.2 ± 7.6 vs. 72.7 ± 17.0 months, p = 0.052); this difference was significant when the analysis was confined to DM patients (19.2 ± 7.6 vs. 65.0 ± 3.5 months, p = 0.032) (figure 2). No evidence of necrotizing myositis or cardiac involvement was observed in one patient with anti-SRP antibody. MAAs were not found to be associated with any particular clinical features of myositis.

**Table 4:** Clinical characteristics of 9 patients positive for anti-p140 antibody

	1	2	3	4	5	6	7	8	9
Age at myositis diagnosis	48	67	18	46	55	21	53	51	53
Sex	F	F	M	M	F	F	F	M	M
Diagnosis	DM	DM	DM	DM	DM	DM	DM	DM	DM
Interstitial lung disease	+	+	-	+	+	-	-	+	+
Rapidly progressive type	No	Yes		No	Yes			Yes	Yes
Muscle weakness	+	+	+	+	+	+	+	+	+
Creatine kinase†	31	20	486	212	930	19487	775	1273	140
Lactate dehydrogenase†	280	407	765	493	437	6916	748	295	249
Abnormal electromyography	+	+	+	+	+	+	+	+	+
Biopsy proven myositis	+	+	+	+	+	+	+	+	+

† Normal range; 20-270 IU/mL for creatine kinase and 100-225 IU/mL for lactate dehydrogenase

DM = dermatomyositis, F = female, M = male

**Table 5:** Associations between myositis autoantibodies and clinical subsets

	Anti-ARS	Anti-p140	Anti-p155/140	Anti-Mi2	Total
	n = 6	n = 9	n = 8	n = 7	n = 49
ILD	5	6	0	0	14
Rapidly progressive ILD	0	4			4
Cancer-associated myositis	3	0	5	1	11
Mortality	2	3	4	0	10
ILD-related	0	3	0	0	3
Cancer-related	1	0	4	0	6

ARS = aminoacyl tRNA synthetase; ILD = interstitial lung disease

## Discussion

The present study shows that anti-p140 (18.4%), anti-p155/140 (16.3%), anti-Mi2 (14.3%), and anti-ARS (12.2%) antibodies are common autoantibodies in Korean patients with inflammatory myositis; the common prevalence of anti-ARS and anti-Mi2 antibodies is consistent with our previous result [20]. Anti-p140, anti-p155/140, and anti-Mi2 antibodies were found exclusively in DM patients as previously reported [9-16], and the first two antibodies were found to be associated with distinctive clinical subsets of myositis; anti-p140 antibody with rapidly progressive ILD and anti-p155/140 antibody with cancer-associated myositis. Furthermore, anti-p155/140 antibody was found to be associated with poor survival in cancer-associated DM patients. Anti-ARS antibody was present in both PM and DM patients and found to be associated with stable or slowly progressive ILD.

The most distinguished findings of this study are the high prevalence of anti-p140 antibody in classic DM patients and the association of this antibody with rapidly progressive ILD independent of CADM. Myositis-associated rapidly progressive ILD has been reported in both classic DM and ADM/CADM patients [5-7,22-25]. Although rapidly progressive ILD often occurs in patients with ADM/CADM [5-7], its true incidence remains unknown due to poor estimates of the proportions of ADM/CADM patients in populations. Previous Japanese studies have reported that anti-p140 antibody is mainly found in CADM rather than classic DM patients [9,12,13], but these studies enrolled few classic DM patients with rapidly progressive ILD to conclusively determine whether anti-p140 antibody is a risk factor of CADM or of rapidly progressive ILD. Recently, Sato et al reported that the RNA helicase encoded by melanoma differentiation-associated gene 5 (MDA5) is an autoantigen recognized by anti-p140 antibody (which currently is called "anti-CADM-140 antibody"), and went on to develop an enzyme-linked immunosorbent assay technique using the recombinant protein [26]. According to their results, anti-p140 antibody is exclusively found in CADM patients with or without rapidly progressive ILD; no significant difference was found between the antibody titers of those with and without ILD. This finding suggests that anti-p140 antibody is primarily associated with the CADM phenotype than rapidly progressive ILD in Japanese patients. On the other hand, our study shows that anti-p140 antibody is one of the most common myositis autoantibodies in Korean patients with classic DM, and that it has a striking association with rapidly progressive ILD. Because no patient in the present study met the ADM/CADM criteria [5], these observations suggest that anti-p140 antibody is primarily associated with rapidly progressive ILD independently of CADM at least in Korean patients.

It has been reported that anti-p155 and/or anti-p155/140 antibodies are associated with cancer-associated myositis [10-12,14-16], and our results support this contention. In addition, we were able to confirm the previously reported lower incidence of ILD among anti-p155/140 positive patients [10-12,14,15]. The poor cancer-related outcome of anti-p155/140 positive patients compared with anti-p155/140 negative patients is a novel finding. However, our study is adopting a small number of patients to conclude with a certainty. We are cautious to claim the prognostic value of this antibody in cancer-associated myositis until it is proven via multivariate analyses that appropriately adjust other prognostic factors. Further studies employing a larger number of cancer-associated myositis patients are warranted to confirm this result and to determine if anti-p155/140 antibody predates or follows the onset of cancer, if its titer correlates with cancer progression, or if certain types of cancers are more prone to develop anti-p155/140 antibody. Another interesting finding was that 3 out of 6 anti-ARS positive patients had cancer-associated myositis, which, however, was not statistically significant. In fact, cancer-associated myositis in the presence of other myositis autoantibodies than anti-p155/140 antibodies (such as anti-ARS or anti-Mi2) has been previously reported [14,27,28]. Although anti-p140 positive patients have been shown to have low prevalence of malignancy [9,12], the presence of other myositis autoantibodies rather than anti-p155/140 antibodies does not seem to rule out the presence of cancer in inflammatory myositis patients.

The limitations of this study are as follows. First, 26 sera were dropped out during consecutive enrollment, which leaves the possibility of selection bias. However, the clinical implications of anti-p140 and anti-p155/140 antibodies observed in this study are unlikely to be affected, because there was only one patient with rapidly progressive ILD and none with malignancy among these 26 patients during 78 ± 59.9 months of follow-up (data not shown). Second, we did not further examine the specificities of myositis autoantibodies beyond immunoprecipitation. Therefore, anti-p140 and anti-p155/140 antibodies detected in the present study may not exactly represent anti-CADM-140 and anti-p155/140 antibodies that have previously been shown to recognize MDA5 [13,26] and transcriptional intermediary factor 1-γ [29], respectively. However, high accuracy of immunoprecipitation to specifically detect anti-p140 or anti-p155/140 antibodies in inflammatory myositis patients has been previously reported [30]. In addition, distinctive clinical features have been demonstrated in association with each myositis autoantibody defined by immunoprecipitation in our study, which is generally in parallel with the results of previous studies [9-16].

## Conclusions

In summary, anti-p140 and anti-p155/140 antibodies were commonly found autoantibodies in Korean patients with inflammatory myositis. Despite the lack of CADM patients in the study subjects, a strong association was observed between anti-p140 antibody and rapidly progressive ILD. Anti-p155/140 antibody was associated with cancer-associated myositis and poor survival, which should be confirmed in a study of larger scale.

## Competing interests

The authors declare that they have no competing interests.

## Authors' contributions

EHK was involved in the acquisition and analysis of clinical data and drafted the manuscript. RN carried out the immunoprecipitation assay. TM helped interpret the immunoprecipitation data and draft the manuscript. JK, LYJ, and LEB helped clinical data acquisition. YWS was involved in the concept and design of the work and helped draft the manuscript. All authors have read and approved the final manuscript.

## Pre-publication history

The pre-publication history for this paper can be accessed here:

http://www.biomedcentral.com/1471-2474/11/223/prepub
